# Improving the Cathodic Biofilm Growth Capabilities of *Kyrpidia spormannii* EA-1 by Undirected Mutagenesis

**DOI:** 10.3390/microorganisms9010077

**Published:** 2020-12-30

**Authors:** Tobias Jung, Max Hackbarth, Harald Horn, Johannes Gescher

**Affiliations:** 1 Department of Applied Biology, Institute for Applied Biosciences, Karlsruhe Institute of Technology (KIT), Fritz-Haber-Weg 2, 76131 Karlsruhe, Germany; tobias.jung@kit.edu; 2 Engler-Bunte-Institut, Chair of Water Chemistry and Water Technology, Karlsruhe Institute of Technology (KIT), Engler-Bunte-Ring 9, 76131 Karlsruhe, Germany; max.hackbarth@kit.edu (M.H.); harald.horn@kit.edu (H.H.); 3 Institute for Biological Interfaces, Karlsruhe Institute of Technology (KIT), Hermann-von-Helmholtz-Platz 1, 76344 Eggenstein-Leopoldshafen, Germany

**Keywords:** microbial electrosynthesis, *Kyrpidia spormannii*, cathodic biofilm, adaptation, mutagenesis, polyhydroxybutyrate, PHB, bioelectrochemistry

## Abstract

The biotechnological usage of carbon dioxide has become a relevant aim for future processes. Microbial electrosynthesis is a rather new technique to energize biological CO_2_ fixation with the advantage to establish a continuous process based on a cathodic biofilm that is supplied with renewable electrical energy as electron and energy source. In this study, the recently characterized cathodic biofilm forming microorganism *Kyrpidia spormannii* strain EA-1 was used in an adaptive laboratory evolution experiment to enhance its cathodic biofilm growth capabilities. At the end of the experiment, the adapted cathodic population exhibited an up to fourfold higher biofilm accumulation rate, as well as faster substratum coverage and a more uniform biofilm morphology compared to the progenitor strain. Genomic variant analysis revealed a genomically heterogeneous population with genetic variations occurring to various extends throughout the community. Via the conducted analysis we identified possible targets for future genetic engineering with the aim to further optimize cathodic growth. Moreover, the results assist in elucidating the underlying processes that enable cathodic biofilm formation.

## 1. Introduction

It is a general interdisciplinary aim to establish carbon dioxide as substrate for applied production processes. In biotechnology, autotrophic organisms are preferentially used as catalysts for CO_2_ valorization. The conversion of carbon dioxide into valuable organic carbon molecules (or biomass) necessitates the availability of suitable reduction equivalents. The energy for this reduction originates conventionally from either chemical or light energy. However, in recent years, electrical energy was elucidated as potential further source of energy and electrons to sustain autotrophy. Processes that are conducted by autotrophic microorganisms interacting with cathodes are subsumed under the term microbial electrosynthesis [1,2,3]. In theory this technology could provide a way for the sustainable storage of renewable energies in the form of organic carbon molecules. There are, however, several drawbacks that this technology has to overcome. First, the number of applicable electroautotrophic biocatalysts is so far rather small and most of them belong to the group of methanogens or acetogens [4,5]. These organisms are often strictly anaerobic and genetic engineering is either not possible or very laborious. Second, this narrow spectrum of organisms also results in a narrow product range with acetate and methane as main end products. Still, some progress has been made concerning different side-products like butyrate, isopropanol, and acetone produced by homoacetogens like *Sporomusa* sp. and *Clostridium* sp. [2,4,6]. Third, the mechanisms of electron import by organisms thriving on cathodes are in most cases fundamentally not understood yet [7,8,9]. One possible electron uptake mechanism is the direct electron import. Here, either cytochromes or conductive pili catalyze electron transfer between the cathode surface and the cytoplasmic membrane of the organisms [10,11]. However, in most described systems electron transfer seems to be mediated either by organic or inorganic molecules [12,13]. The spectrum of these electron shuttles can range from inorganic electron shuttling molecules such as sulfur-, iron-, or other metal-ions to organic molecules such as ferredoxins, flavins, or other redox-active molecules. The latter group is most often actively secreted or released during cell lysis [12,14]. Among the inorganic electron shuttles, hydrogen is one of the most controversially discussed as its formation can be catalyzed by microorganisms that interact with the cathode but can also be the result of abiotic proton reduction on the cathode surface. Hence, it is a matter of debate whether there is in some cases a difference between electrosynthesis and chemolithoautotrophic growth with hydrogen as electron donor and energy source [9].

Methanogens or acetogens thriving on cathodes use carbon dioxide as electron acceptor. The rather narrow difference in the redox potential between the electrode and the electron acceptor CO_2_ limits growth and also the possibility to genetically steer the metabolism of the organisms towards valuable end products. Studies on aerobic microorganisms that grew on the surfaces of microbial fuel cell cathodes provided evidence that microorganisms can catalyze oxygen reduction using the cathode as electron donor [15]. Using oxygen as electron acceptor increases the potential energy supply for the organisms. Higher energy availability could provide the foundation for the genetic engineering of the organisms towards the production of more valuable end products derived from electrons and CO_2_. Still, the number of autotrophic model organisms that can thrive aerobically on a cathode surface is rather narrow. The reason might be that the abiotic reaction of the cathode surface with oxygen will lead to the production of reactive oxygen species, which will hamper microbial growth. Bause and colleagues studied chemolithoautotrophic growth of the mesophilic knallgas bacterium *Cupriavidus necator* on cathodes poised to potentials that lead to the production of hydrogen as electron donor [16]. The organism covered not more than 35% of the cathode surface even under the identified optimal conditions. The authors discuss that the reason for the absence of confluent growth might be reactive oxygen species. In recent studies, *Kyrpidia spormannii* was presented as a new thermoacidophilic, obligate aerobic knallgas bacterium, which is able to grow electroautotrophically and forms comparatively thick and confluent biofilms on a cathode as sole electron and energy source [17,18]. These findings, together with its ability to use CO_2_ as a carbon source even if this is part of an unpurified flue gas, renders it an interesting new biocatalyst for microbial electrosynthesis processes [19]. In addition, *K. spormannii* is a natural producer of polyhydroxybutyrate (PHB), a biotechnologically interesting product that can serve as a bio-based and biodegradable polymer for the production of bioplastics. Still, the industrial application of the strain necessitates an improvement of the space-time-yields of biomass formation on the cathode surfaces, while biomass harvesting could be easily realized using mechanical sheering of the biofilm form the substratum. This necessity was the motivation for the here described adaptive laboratory evolution experiment. We observed how biofilm growth on cathodes improved over several transfers and elucidated the potential reasons for this improvement by resequencing the strain and analyzing the genetic variations.

## 2. Materials and Methods

### 2.1. Liquid Cultivation of Kyrpidia spormannii

Liquid cultivation of *K. spormannii* under heterotrophic conditions was conducted in a modified R2A medium (yeast extract 5 g L^−1^, tryptone 1 g L^−1^, casamino acids 0.5 g L^−1^, sodium pyruvate 0.5 g L^−1^, MgSO_4_·7 H_2_O 0.05 g L^−1^, K_2_HPO_4_ 0.1 g L^−1^, MOPS 10 mM pH 6) at 60 °C without agitation. The cultivation with the inorganic electron donors H_2_ or elemental sulfur (S^0^) was carried out in laboratory glass bottles (Schott, Mainz, Germany), using ES minimal medium according to [19]. The bottles were closed with rubber stoppers, degassed by repeated cycles of vacuum and N_2_ purging and autoclaved afterwards. Cultivation was carried out at 60 °C. For hydrogenotrophic growth conditions, the gas phase was exchanged with H_2_/CO_2_/O_2_ (80:15:5% *v*/*v*) (1 bar overpressure) at the beginning of the cultivation and renewed every 5 days. For cultivation with elemental sulfur, 1 g L^−1^ S^0^ was added to the medium after autoclaving and a gas phase of CO_2_/O_2_ (95:5% *v*/*v*) was established. S^0^ was sterilized by tyndallization at 100 °C for 3 h on three consecutive days. Samples for pH, OD_600_, and chromatographic analyses were taken at several time points throughout the growth experiments.

### 2.2. Bioelectrochemical Cultivation

**Figure A1:**
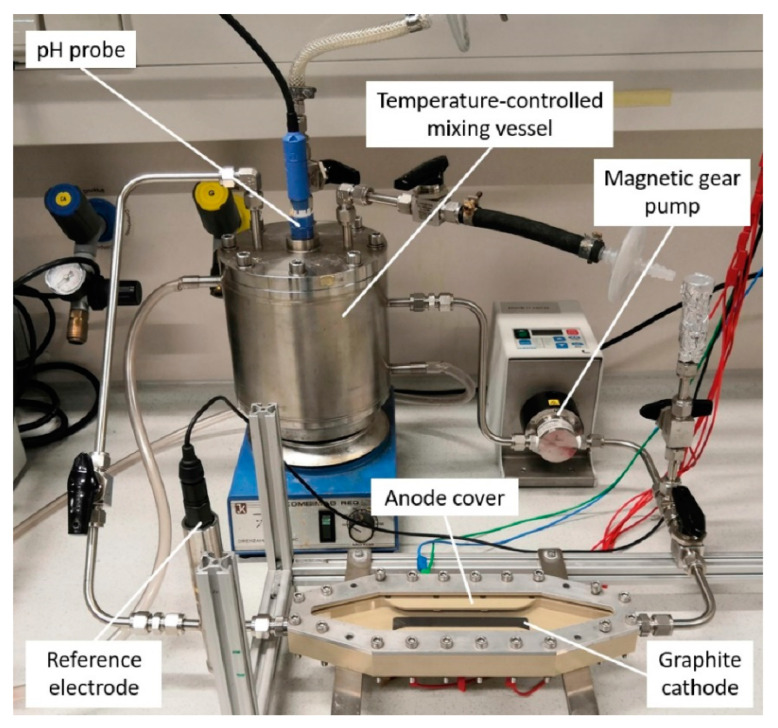
Photographic image of the flow-cell setup utilized in this study.

The bioelectrochemical cultivation was performed using a custom-made bioelectrochemical system (BES) flow-cell setup with its general structure and detailed operational conditions published elsewhere [18]. An image of the setup is presented in Appendix A Figure A1. In short, a heterotrophically grown pre-culture of *K. spormannii* was washed two times with ES medium before inoculating the sterile flow-cell setup with an OD_600_ of 0.1. The flow-cells were operated at 60 °C for 10 or 28 days, respectively. The supply with carbon source and electron acceptor was realized by pressurizing the system (1.5 bar) using a gas mixture of CO_2_ and O_2_ (99.5:0.5% *v*/*v*). The gas phase was replaced every 2–3 days. The liquid phase in the double jacket boiler was stirred at 300 rpm and the flow rate of the magnetic gear pump was adjusted to 100 mL min^−1^. Plain graphite (MR40; Müller und Rössner, Troisdorf-Bergheim, Germany) was used as cathode material. The counter electrodes were made of iridium-tantalum coated titanium (Platinode^®^—MMO Anode 177; umicore, Schwäbisch Gmünd, Germany). To ensure a slow electrode polarization, cultivations started with a linear sweep voltammetry (LSV) experiment from 0 to −500 mV vs. standard hydrogen electrode (SHE) using step sizes of 2 mV over a period of 18 h. The potential of −500 mV vs. SHE was held using chronoamperometric (CA) mode for the rest of the experiment. The optical coherence tomography (OCT) datasets were generated using a Ganymede II OCT device equipped with a LSM04 lens (Thorlabs, Dachau, Germany) as described by [18]. The analysis was conducted as described elsewhere [18,20,21].

### 2.3. UV-Assisted Cathodic Adaptation

**Figure 1:**
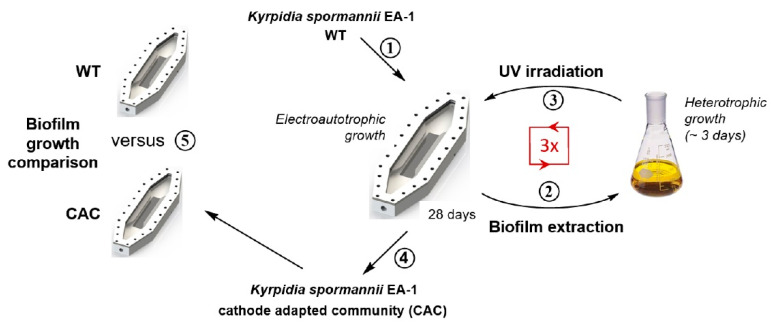
Scheme of UV-assisted cathodic adaptation experiment. (**1**) Cultivation of *K. spormannii* EA-1 wild type (WT) in the flow cells under electroautotrophic growth conditions. (**2**) Biofilm extraction and heterotrophic cultivation. (**3**) UV irradiation of the inoculum and transfer to the next flow cell cultivation. Steps 2 and 3 were repeated three times in total. (**4**) Extraction of the cathode adapted community (CAC). (**5**) Comparison of the WT and CAC in terms of electroautotrophic biofilm growth.

In order to achieve the adaptation of the *K. spormannii* EA-1 wildtype (WT) to the electroautotrophic growth conditions, the strain was transferred four times in sterile BES flow cells and cultivated for 28 days (see Figure 1). Afterwards the biofilm was extracted, cells were grown heterotrophically and, to increase the mutation rate, an ultraviolet light mutagenesis was performed before each of the following flow-cell inoculations. To this end, half of the inoculum for the flow-cells was poured into a petri dish and placed 15 cm under a UV lamp (VL-4LC, Vilber Lourmant, Collégien, France). The cells were irradiated with UV-C light (λ_254_ nm) for 3 s under slight shaking. Then, the flow cell was inoculated with both, irradiated and unirradiated cells. Bioelectrochemical growth was performed as described before, except that the flow cell lid was covered for at least 7 days. The biofilm-extraction- and UV-irradiation-cycles were repeated for three times in order to end up with four individual periods of 28 days of electroautotrophic growth. In order to evaluate the cathodic adaptation of the adapted community, the cathodic biofilm growth in the flow cells was compared to the WT strain for 10 days. 

### 2.4. Genome Sequencing and Variant Detection

Genomic DNA was isolated from *K. spormannii* culture after laboratory evolution, using the Wizard^®^ Genomic DNA Purification Kit (Promega, Madison, WI, USA) according to the manufacturer’s instructions for Gram positive bacteria. Sequencing was conducted by IMGM Laboratories GmbH (Martinsried, Germany) on an Illumina MiSeq platform using 2 × 150 bp paired-end (PE) reads. The DNA library was generated with the NEBNext^®^ Ultra™ II FS DNA library preparation kit. Sequencing Signal processing and de-multiplexing were conducted by IMGM Laboratories using the MiSeq Reporter 2.5.1.3 software (Illumina, Berlin, Germany). Further bioinformatic analysis was conducted using the CLC Genomics Workbench v. 12.0 (Qiagen, Aarhus, Denmark). Adapter sequences were trimmed, low quality sequences were removed (quality score < 0.05) and the resulting sequences were mapped to the reference genome of *K. spormannii* (Genbank sequence CP024955) [22]. Mutations in the genome of the adapted community were detected using the Low Frequency Variation Detection plugin of the CLC Genomics Workbench. To this end, standard parameters were chosen, except for the required significance (1%) and the minimum frequency (10%).

### 2.5. Ion Exchange Chromatography (IC)

IC analysis was conducted using the ion chromatograph Dionex™ ICS-1100, equipped with the anion exchange column Dionex™ IonPac™ AS9-HC (2 × 250 mm) and the precolumn Dionex™ IonPac™ AG9-HC (2 × 50 mm) (Thermo Fisher, Waltham, MA, USA). A 0.9 mM sodium carbonate buffer was used as eluent. The DS6 conductivity detector was used, as well as the suppressor AERS 500 (2 mm), operated at 12 mV, to reduce basic conductivity of the eluent (both Thermo Fisher, Waltham, MA, USA). The setup was operated at 0.25 mL min^−1^ and 30 °C. Prior to injection, the samples were filtered with a 0.2 µm PTFE syringe filter (VWR, Radnor, PA, USA) to remove cells and sulfur particles. For SO_4_^2−^ quantification, sodium sulfate was used as calibration standard.

### 2.6. Blast- and Protein Domain Analyses

Sequence homology analyses were performed using BLASTX analysis on the servers of the National Center for Biotechnology Information NCBI (Rockville Pike, Bethesda, MD, USA) using the non-redundant protein sequences (nr) database. The analysis of conserved protein domains was also carried out on the servers of NCBI using the conserved domain database (CDD) [23].

## 3. Results and Discussion

### 3.1. Cathodic Biofilm Growth

**Figure 2:**
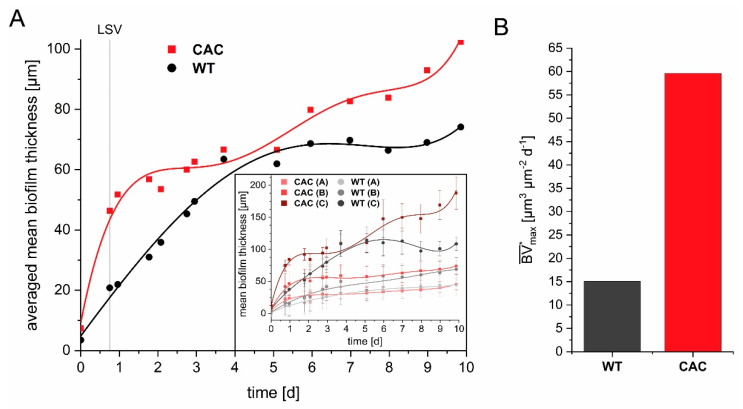
Course of the determined averaged mean biofilm thickness (**A**) and the maximum biofilm accumulation rates ${\bar{\mathrm{BV}}}_{\max}^{*}$ of CAC and WT (**B**) during flow cell cultivation. The inset in A depicts the mean biofilm thickness including standard deviations for the individual measuring points A, B, and C, located in the front, middle and back part of the cathode. The data points were fitted using a polynomial fit of 5th order with R^2^ > 0.96. ${\bar{\mathrm{BV}}}_{\max}^{*}$ was determined as described in [18]. The time point of the linear sweep voltammetry (LSV) end is marked.

*K. spormannii* wild type (WT) was adapted to cathodic growth over a time period of four months. We assumed that the wild type strain evolved within this time period to several different strains with specific adaptations towards individual process conditions that simultaneously occur in the flow cell reactor. At the end of the last selection cycle, we scraped of the biomass and used this sample of the cathode adapted community (CAC) as inoculum for growth under heterotrophic conditions. This CAC-culture as well as an heterotrophically grown wild type culture were used to comparatively analyze growth within the cathodic flow-cell setup. Biofilm 3D visualizations were routinely performed using optical coherence tomography (OCT). The processed OCT data sets showed an averaged mean biofilm thickness of approximately 102 µm after 10 days of cultivation, an improvement of 28% compared to the WT (Figure 2A). However, the biggest difference between CAC and the WT in the cathodic growth was the maximum biofilm accumulation rate ${\bar{BV}}_{\max}^{*}$ (measured between inoculation and day three), that was roughly four times higher for the CAC-culture (Figure 2B).

**Figure 3:**
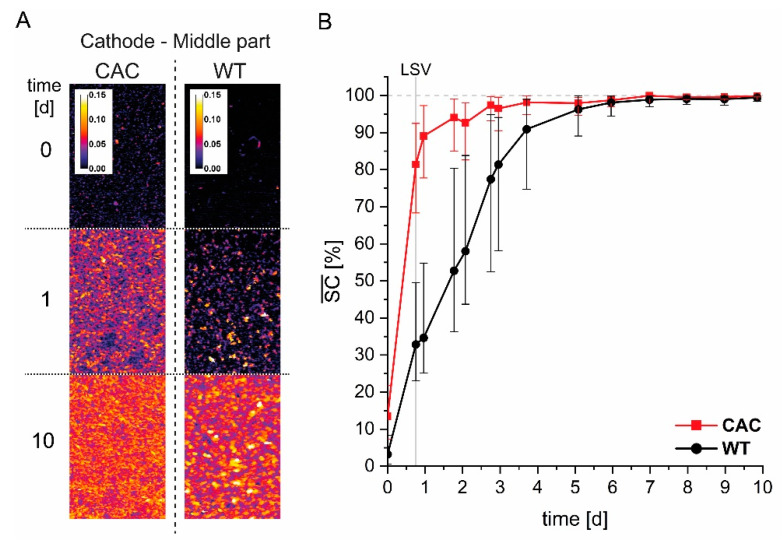
Macroscopic evaluation of the *K. spormannii* biofilms depicted in height maps (**A**) and the course of the mean substratum coverage $\bar{\mathrm{SC}}$ (**B**) for WT and CAC. The height maps were generated using optical coherence tomography (OCT) data set recordings of the middle cathode position B at three time points during the cultivation. The calibration bar for the height maps is given in [mm]. The mean substratum coverage $\bar{\mathrm{SC}}$ was calculated by averaging the corresponding substratum coverage SC values. Error bars depict the minimum and maximum SC -values at the respective time points. The time point of the LSV end is marked.

**Figure A2:**
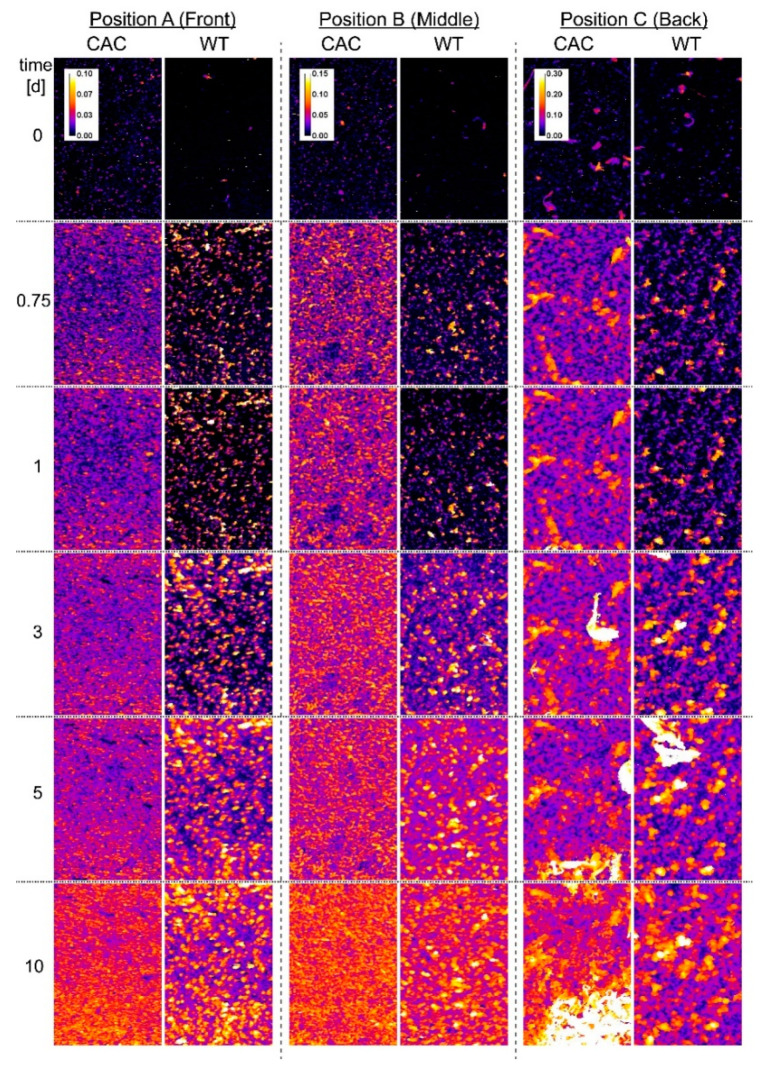
Height maps of the biofilm of CAC and the WT during flow cell cultivation. The height maps for the individual measuring points A, B, and C, located in the front, middle and back part of the cathode, respectively, are displayed (see [18] for further information). Note the varying legends for the different measuring points, given in mm.

**Figure A3:**
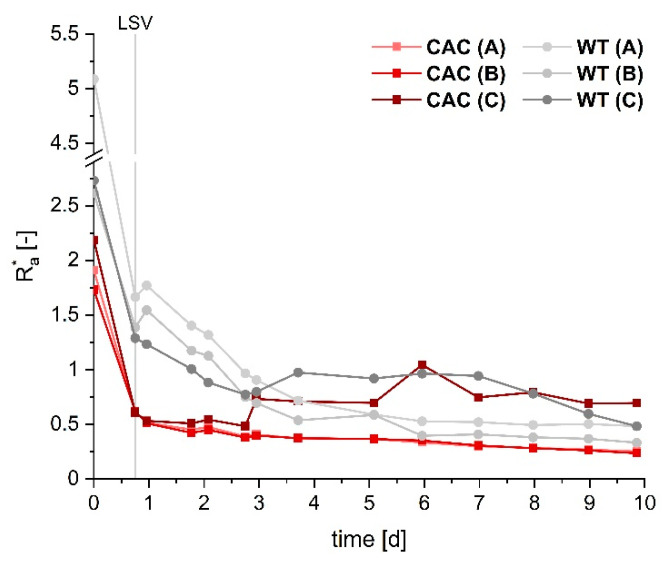
Roughness coefficient $R_{a}^{*}$ for CAC and the wild type (WT) during flow cell cultivation for the individual measuring points A, B, and C, located in the front, middle and back part of the cathode, respectively (see [18] for further information). The time point of the LSV end is marked.

In order to evaluate the macroscopic biofilm morphology, height maps of the biofilm were created for nearly each of the conducted C-scans at the front-, middle-, and back-part of the cathode (Appendix A Figure A2). Figure 3A depicts a selection of height maps of the middle part of the cathode for both strains at different cultivation time points. By evaluating the height maps and the corresponding values for the mean substratum (cathode) coverage $\bar{SC}$ (Figure 3B) and the biofilm roughness coefficient $R_{a}^{*}$ (Appendix A Figure A3), two main differences in biofilm morphology became obvious. First, an almost complete coverage of the cathode (>95% mean coverage) was achieved after ~2.8 days for CAC, while this took twice as long for the WT. Second, the WT did form a biofilm with an evidently rougher surface structure, forming tower-like structures, whereas CAC formed a more homogenous biofilm. The graphs of $\bar{SC}$ as well as $R_{a}^{*}$ also corroborate the visual images of the height maps.

The observed changes in substratum coverage and biofilm morphology most probably target two main obstacles for cathodic growth under the chosen process conditions which are the production of reactive oxygen species and access to the electron donor. We describe an oxic microbial electrosynthesis process. Hence, oxygen can be abiotically reduced at the cathode surface. This process will lead to the production of reactive oxygen species which will hamper growth of the organisms. Fast substratum coverage and respiration-assisted oxygen depletion will reduce this unwanted abiotic reaction and lead to better growth conditions. This insulation-effect of aerobically growing electroautotrophic biofilms was already postulated for *K. spormannii* recently [18]. On the other hand, rather uniform growth on the electron acceptor will lead to an on average reduced distance between the organisms and the electron surface which will have a positive impact on electron donor availability.

### 3.2. Comparative Genome Variation Analysis

**Figure 4:**
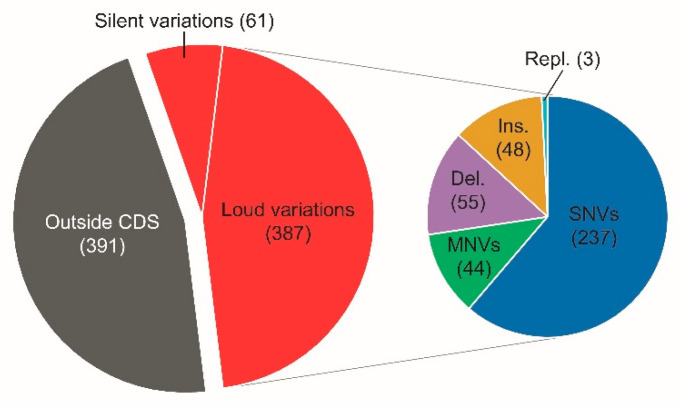
Distribution of the identified 838 variations occurring at a frequency of more than 10% in the population of CAC compared to the WT. SNVs—single nucleotide variations; MNVs—multiple nucleotide variations; Del.—deletions; Ins.—insertions: Repl.—Replacements.

As comparative cultivation under electroautotrophic conditions showed an improved cathodic biofilm growth of the adapted community, we aimed to elucidate the underlying molecular differences using resequencing of the community and subsequent comparison to the wild type genome. It was possible to map 38,058,785 reads to the wildtype genome, resulting in a reasonable base coverage of 1496 (±290). By setting the threshold of the variant detection to a minimum frequency of 10%, the number of results was kept lower and more condensed to detect more potentially significant variations that have established in the population. In total, 839 variations could be detected in the CAC-culture. Around 54% of all variations occurred within coding regions, of which 14% were silent mutations (Figure 4). SNVs (single nucleotide variations) account for a large number of the loud mutations (60%). MNVs (multiple nucleotide variations), deletions and insertions together account for a further 39%, whereas replacements only occurred to a minor portion.

Variations that occurred in over 50% of the population include three loud SNVs, two silent and one mutation outside of a coding sequence. Table 1 displays a more detailed overview of these variations. Even though silent mutations, as well as variations in non-coding or intergenic sequences have recently been proven to be capable of impacting protein expression levels due to, e.g., alterations in regulatory DNA sequences or mRNA stability, respectively, they were not the focus of this study [24,25,26]. The reason is that there is so far no genetic system for *K. spormannii* to study these genomic variations more intensively in vivo. Hence, at this point it would even be impossible to only speculate on the function of these mutations. Consequently, the focus of this study was to analyze the three loud mutations occurring in the cathode adapted community of *K. spormannii*. 

#### 3.2.1. Adaption to Oxidative Stress

The nucleotide exchange 2708G > T in the coding sequence of *mfd* (*CVV_00395*) results in 55.3% of the population in the amino acid change G903V. This gene is annotated as *mfd* (*transcription-repair coupling factor*), which could also be corroborated by further blast-analyses (Table A1 in Appendix A). The protein Mfd (mutation frequency decline) is part of a DNA repair machinery. It detects and dissociates a stalled RNA polymerase (RNAP) from the DNA [27]. This happens especially when non-coding DNA lesions (like pyrimidine dimers) have formed due to, e.g., UV light or oxidative stress. As a result, Mfd recruits the DNA repair complex UvrABC, which is able to repair the DNA lesion by nucleotide excision repair [27,28]. This mutation could be a result of the repetitive UV-treatment that was conducted during the adaptation phase. It could also be the result of reactive oxygen species formation on the cathode of the bioelectrochemical system. This will likely take place at least as long as the cathode is not fully covered with biofilm and dissolved oxygen can reach to the cathode surface before it is consumed by *K. spormannii* cells (see above). Unfortunately, the position of the amino acid exchange is not instructive per se. Hence, it is so far not possible to hypothesize how the mutation might affect the activity of the protein.

The nucleotide exchange 410G > T in the coding sequence of the gene *CVV_06825* occurred in 99.8% of the population and led to the amino acid change C137F. The genome annotation, as well as further analyses led to the assumption that CVV_06825 is part of the Fur protein family, which consists mainly of a number of regulators for the Fe^2+^-, Zn^2+^- or Mn^2+^-uptake (Table A1 in Appendix A). The highest homologies could be identified to the Fur-protein PerR, a highly conserved H_2_O_2_- and NO-sensor protein in gram positive bacteria. In *Bacillus subtilis*, its regulon includes—besides *perR* itself—further genes involved in the defense of oxidative stress (*katA*, *ahpCF* and *mrgA*), metal ion homeostasis (*zosA*, *fur*, *hemAXCDBL*), and the production of surfactants (*srfA*) [29]. 

**Figure A4:**
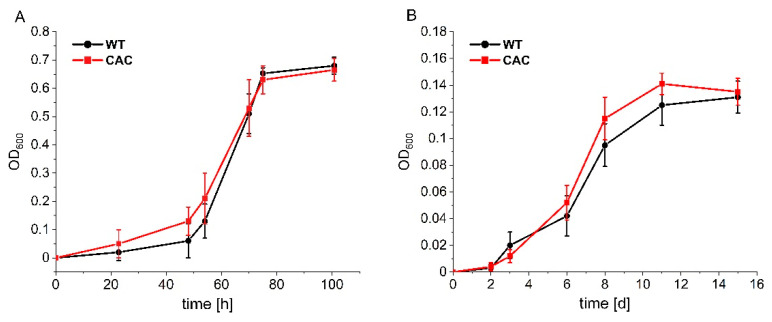
Growth curve of *K. spormannii* EA-1 CAC and the wild type (WT) under heterotrophic (**A**) and hydrogenotrophic (**B**) growth conditions using RR2A medium and ES-medium supplemented with H_2_, CO_2_, and O_2_. Error bars represent the standard deviation of a triplicate.

The variant in the CAC-population shows an exchange of a cysteine for a phenylalanine (C137F). This cysteine is one the four cysteines of two conserved -CXXC- motifs at the C-terminus of the protein, which are essential for the stable coordination of a structurally important Zn^2+^-atom [30]. The binding of Zn^2+^ is essential for the dimerization of two PerR monomers, and thus the structure and ultimately the function of PerR as a DNA binding H_2_O_2_ sensor protein [31]. Structural studies of PerR in *B. subtilis* have shown that the exchange of the corresponding cysteine C139 to a serine leads to the loss of the repressor function [30]. Because of its hydrophobic nature, phenylalanine is—similarly to serine—no typical metal ligand in proteins. Hence, we hypothesize that the mutation either led to a complete loss of the repressor function due to Zn^2+^ loss, or to an unstable Zn^2+^ binding capacity and consequently an impaired repressor activity [32]. The latter scenario is supported by the fact that PerR mutants of *B. subtilis* are more resistant to H_2_O_2_. Nevertheless, the *B. subtilis* mutant is also characterized by slower growth, an observation that could not be made with the CAC-culture in this study, neither under heterotrophic nor hydrogenotrophic growth conditions (Figure A4 in Appendix A) [33]. Still, a decreased repressor function and a consequently higher expression of proteins that cope with oxidative stress, could be a specific adaptation to electroautotrophic growth under oxic conditions. This could have also led to the observed faster complete cathode coverage.

#### 3.2.2. Sulfur-Species as Potential Electron Donor

**Figure 5:**
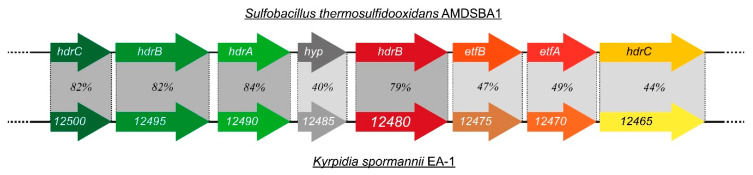
Schematic representation of the *hdr*-like gene cluster of *Sulfobacillus thermosulfidooxidans* AMDSBA1 with the respective sequence identity (in %) of the corresponding encoded proteins of *K. spormannii* EA-1. The sequence coverage was over 90% for all investigated proteins. Data on *S. thermosulfidooxidans* was obtained from [35].

In 99.8% of the population of the CAC a variation was detected that led to the amino acid exchange V233G in CVV_12480, an annotated Fe-S binding protein in the genome of *K. spormannii*. The analysis of preserved protein domains revealed a potential role in energy conservation as part of the succinate dehydrogenase/fumarate reductase complex or as subunit of a CoB-CoM heterodisulfide reductase (HDR) complex (Appendix A Table A1). The HDR-complex is particularly important for methanogenic archaea as the reductase complex of the heterodisulfide CoB-S-S-CoM. However, recent studies point out that HDR-like proteins are also present in acidophilic sulfur oxidizing bacteria. Their HDR-complex seems to play a crucial role in the intracellular oxidation of protein-bound reduced sulfane–sulfur compounds (RSS_n_H) to sulfite [34,35]. Further blast analyses of the *CVV_12480*-surrounding genes showed high sequence identities to various genes of heterodisulfide reductase (*hdr*)-like complex genes of *Kyrpidia tusciae* and different *Sulfobacillus* sp., respectively (not shown). In fact, all gene-encoded proteins of the analyzed gene cluster of *K. spormannii* (*CVV_12500*-*12465*) have a rather high identity to the described *hdr*-like gene cluster of *Sulfobacillus thermosulfidooxidans* AMDSBA1 (Figure 5). Similar gene clusters could also be found in other acidophilic sulfur oxidizers such as *Acidithiobacillus* sp. or *Aquifex aeolicus* [36,37].

**Figure 6:**
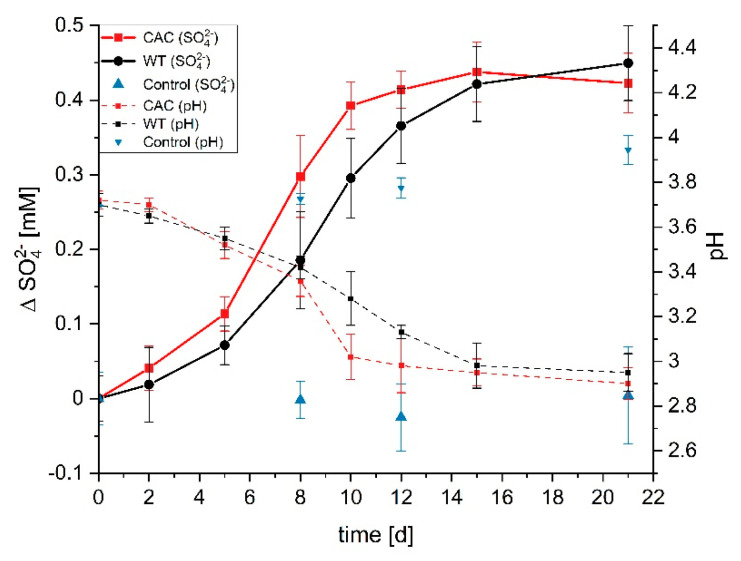
Growth of *Kyrpidia spormannii* WT and CAC in laboratory glass bottles using S^0^ as electron and energy source. The change in sulfate concentration compared t_0_ (Δ SO_4_^2−^) and pH are displayed. The control was an abiotic experiment. Error bars represent the standard deviation of a triplicate.

So far, the ability to oxidize sulfur species was not described for *K. spormannii*. Therefore, growth experiments of the wild type and CAC under lithoautotrophic growth conditions were performed using elemental sulfur (S^0^) as electron and energy source, CO_2_ as carbon source, and O_2_ as electron acceptor. The simultaneous accumulation of SO_4_^2−^, together with a decrease in pH in the course of the experiment are evidence for the metabolic activity and growth of the wild type, as well as CAC on S^0^ (Figure 6). Although CAC oxidizes S^0^ on average faster compared to the wild type, the results are not statistically significant.

Nevertheless, a variety of other electrotrophically growing microorganisms like *Acidithiobacillus ferrooxidans*, *Thiobacillus denitrificans,* and *Sulfurimonas denitrificans* possess an HDR-like complex and are able to oxidize inorganic sulfur species [38,39]. This clustering of sulfur oxidizers within the group of potential electrotrophs could suggest that reduced sulfur species can be used as electron shuttles between cathode and organism or that the mechanism for oxidation of inorganic sulfur can also be used for the uptake of electrons from cathode surfaces. Nevertheless, a direct abiotic reduction of dissolved SO_4_^2−^ by the cathode seems unlikely under typical electrotrophic growth condition [40,41]. Nevertheless, as a variety of protein-bound sulfane–sulfur groups are putative substrates for the HDR complex in the cytoplasm, different so far unnoticed reduced sulfur-oxide or -carbon compounds might be initial extracellular substrates that form at the cathode surface [35,42]. Even though there is so far no direct proof for the involvement of these substances in microbial electrosynthesis processes, it was shown that polysulfide-compounds or cysteine can be important for interspecies electron transport, indicating electron shuttle abilities [12,43]. However, it has not yet been possible to detect any of these compounds in the bulk medium of *K. spormannii*. Still, as it forms biofilms on the cathode surface, an involvement of cell-surface bound cysteine groups as in a number of different organisms, such as *Listeria monocytogenes* seems more plausible, but it has not yet been tested [44,45]. The assumption that the HDR-like complex could play a role under electroautotrophic conditions is corroborated by a recent study, in which the authors elucidated that the genes *CVV_12500-12465* are upregulated under electroautotrophic conditions compared to hydrogenotrophic and heterotrophic conditions, with log2-fold changes of 0.3–1.7 and 3–4.5 for the different genes, respectively [19].

## 4. Conclusions

The adaptation of *Kyrpidia spormannii* EA-1 to electroautotrophic growth conditions was particularly evident in an approximately fourfold higher biofilm accumulation rate, a faster complete cathode coverage and the formation of a more homogeneous biofilm. These improvements are key for the envisioned industrial application of the strain as the desired end product PHB will be purified from harvested biomass. Hence, a fast substratum coverage and biomass accumulation will directly correlate with an increased space-time-yield for PHB production. Certainly, we will have to define in future studies the trigger for PHB production in *K. spormannii* but could already provide evidence that cathodic growth itself can trigger to some extend the production of this biopolymer. The conducted genomic variation analysis of CAC showed the occurrence and establishment of a number of different variations with varying frequencies. The most frequent variations indicate an adaptation to the prevailing oxidative stress, a fact that has been insufficiently studied in previous studies on oxic microbial electrosynthesis systems. As it seems to have a negative impact on biofilm formation, as well as on the overall coulombic efficiency of the process, further studies should address this problem more thoroughly. Furthermore, a variation of a potential HDR-like complex protein could be identified, whereupon it was possible to prove the growth of *K. spormannii* with elemental sulfur. Although the H_2_-dependent electron uptake has been postulated so far for *K. spormannii* and the involvement of sulfur compounds was not shown to play a role in electron uptake mechanisms of microbial electrosynthesis processes, this discovery could be a potential starting point for future research on the underlying processes of microbial electron uptake.

## Figures and Tables

**Table 1:** Results of the variations with a frequency of more than 50% in the Illumina sequencing data of CAC. The numbering of nucleotides and the annotations refer to the sequenced and annotated genome of *K. spormannii* EA-1. Variations that are further referred to in this study are marked in grey. CR—coding region; AA—amino acid.

NT#	Annotation	Type	Variation	CR-Change	AA-Change	Frequency [%]
**71257**	*mfd*	SNV	G → T	2708G > T	G903V	55.3
**1352442**	*CVV65_06825*	SNV	G → T	410G > T	C137F	99.8
**1515328**	*-*	Ins.	- → GG	-	-	90.8
**2495265**	*CVV65_12480*	SNV	A → C	932T > G	V311G	99.8
**2838678**	*CVV65_14005*	SNV	T → C	822A > G	-	99.4
**3335370**	*CVV65_16360*	SNV	G → T	1425G > T	-	99.8

## Data Availability

Data is contained within the article.

## Appendix A

**Table A1:** Results of blast- and protein domain analyses for the genes that were analyzed in more detail during this study. The first 2–3 results are displayed, respectively. Conserved protein domains are depicted with the corresponding description and accession number.

***mfd*** **(*CVV_00395*)**			
**Blast-Analysis:**			
**Description**	**Next Relatives**	**e-Value**	**Identity [%]**
transcription-repair coupling factor	*Kyrpidia tusciae*	0	96.74
	*Effusibacillus pohliae*	0	62.30
**Protein Domain Analysis:**			
Protein domain (accession-No.)	Description		e-value
Mfd (COG1197)	transcription-repair coupling factor (superfamily II helicase)		0
Mfd (TIGR00580)	transcription-repair coupling factor (mfd)		0
***CVV_06825***			
**Blast-Analysis:**			
Description	Next relatives	e-value	Identity [%]
transcriptional repressor	*Kyrpidia tusciae*	0	98.59
peroxide-responsive transcriptional repressor PerR	*Halobacillus halophilus*	0	60.74
**Protein Domain Analysis:**			
Protein domain (accession-No.)	Description		e-value
Fur (COG0735)	Fe^2+^ or Zn^2+^ uptake regulation protein		1.6 × e^−33^
Fur_like (cd0753)	Ferric uptake regulator (Fur) and related metalloregulatory proteins; DNA-binding repressors and activators		3.2 × e^−32^
***CVV_12480***			
**Blast-Analysis:**			
Description	Next relatives	e-value	Identity [%]
(Fe-S)-binding protein	*Kyrpidia tusciae*	0	99.01
	*Sulfobacillus thermosulfidooxidans*	0	79.11
**Protein Domain Analysis:**			
Protein domain (accession-No.)	Description		e-value
GlpC (COG0247)	Fe-S oxidoreductase [Energy production and conversion]		6.4 × e^−32^
PRK06259	succinate dehydrogenase/fumarate reductase iron–sulfur subunit		3.4 × e^−29^
CoB_CoM_SS_C (TIGR03290)	CoB-CoM heterodisulfide reductase, subunit C; The last step in methanogenesis leaves two coenzymes of methanogenesis, CoM and CoB; Similar enzyme complex subunits are found in various other species, but likely act on a different substrate		2.9 × e^−14^
